# The efficacy of immune checkpoint inhibitors on low PD‐L1 cervical cancer: A meta‐analysis

**DOI:** 10.1002/hsr2.2069

**Published:** 2024-05-02

**Authors:** Wutao Chen, Nan Zhang, Zhihong He, Qing Li, You Wang, Weihua Lou, Wen Di

**Affiliations:** ^1^ Shanghai Key Laboratory of Gynecologic Oncology, School of Medicine, Renji Hospital Shanghai Jiaotong University Shanghai China; ^2^ Department of Obstetrics and Gynecology, School of Medicine, Renji Hospital Shanghai Jiaotong University Shanghai China; ^3^ State Key Laboratory of Systems Medicine for Cancer Shanghai China

**Keywords:** cervical cancer, chemotherapy, immune checkpoint inhibitor, immunotherapy, meta‐analysis, prognosis

## Abstract

**Background and Aims:**

The effectiveness of immune checkpoint inhibitors (ICIs) in low programmed death ligand 1 (PD‐L1) expression in cervical cancer (CC) patients remains unknown. We aimed to evaluate the efficacy of ICIs in low PD‐L1 expression CC patients.

**Methods:**

The study is an individual patient data (IPD)‐based meta‐analysis. IPD were compiled through KMSubtraction and IPDfromKM methodologies from high‐quality randomized clinical trials and single‐arm studies which reported overall survival (OS) or progression‐free survival (PFS) stratified by PD‐L1 expression. Kaplan−Meier curves and Cox regression analysis were employed to evaluate the survival benefits of ICIs.

**Results:**

A total of eight studies and 1110 cases were included in the analysis. Within the low PD‐L1 expression subgroup, ICI combination therapy, but not ICI monotherapy, demonstrated significant OS benefits over non‐ICI treatment (hazard ratio [HR] = 0.61, 95% confidence interval [CI]: 0.36−1.04, *p* = 0.06). Concerning PFS, ICI monotherapy was associated with a negative effect compared to non‐ICI treatment (HR = 4.59, 95% CI: 2.32−9.07, *p* < 0.001). Notably, both OS and PFS outcomes were unfavorable for ICI monotherapy compared to both non‐ICI and ICI combination therapy in the combined positive score <1 subgroup (OS: HR = 2.60, 95% CI: 1.31−5.16, *p* = 0.008; PFS: HR = 7.59, 95% CI: 3.53−16.31, *p* < 0.001).

**Conclusion:**

In patients with CC and low PD‐L1 expression, ICI monotherapy may not be considered as the optimal treatment strategy when compared to non‐ICI treatment or ICI combination therapy.

**Registration:**

CRD42023395103.

## 1 INTRODUCTION

Cervical cancer (CC) stands as a prominent and concerning health issue affecting the well‐being of women, representing the second leading cause of cancer‐related mortality in women aged 20−39.^1^ Locally advanced, metastatic, or recurrent CC has long been a substantial challenge for healthcare providers. Concurrent platinum‐based chemoradiotherapy was the first‐line therapy for advanced CC, which conferred patients a significant overall survival (OS) benefit compared to radiotherapy alone.^2^ The integration of the antiangiogenic agent bevacizumab into the treatment regimen has further advanced the field, contributing to an extended 3.7‐month median OS time when combined with chemotherapy.^3^

The landscape of CC treatment has witnessed a transformative shift with the advent of immunotherapy, notably through the exploration of immune checkpoint inhibitors (ICIs) either as monotherapy or in combination with chemotherapy.^4^ Notably, the use of pembrolizumab monotherapy was first approved to be used in programmed death ligand 1 (PD‐L1) positive advanced CC by FDA based on the result of KEYNOTE‐158 in 2018.^5^ Moreover, findings from the KEYNOTE‐826 trial have underscored the benefits of pembrolizumab combined with platinum‐based chemotherapy, with or without bevacizumab, manifesting in prolonged progression‐free survival (PFS) and OS among metastatic CC patients within the PD‐L1 CPS (combined positive rate) ≥1 population.^6^ Recent insights from the GOG‐3016 study revealed a notable OS advantage of cemiplimab over chemotherapy, specifically within the TPS (tumor proportion score) ≥1% subgroup (hazard ratio [HR] = 0.70).^7^ Equally, the CheckMate‐358 trial reported a promising objective response rate of 26.3% for patients with recurrent/metastatic CC undergoing nivolumab treatment.^8^

However, while these studies have significantly advanced our understanding, a noteworthy gap remains in our knowledge—particularly pertaining to the efficacy of PD‐1/PD‐L1 inhibitors within the low PD‐L1 expression subgroup (CPS < 1 or TPS < 1%). Limited data generated from the GOG‐3016 study indicated that some PD‐L1 negative patients might benefit from the administration of cemiplimab in the setting of recurrent CC.^7^ Owing to the relatively limited sample size in this subgroup, the question of the optimal treatment for PD‐L1 negative patients continues to pose a challenge. To bridge this gap, we conducted a meta‐analysis utilizing individual patient data (IPD) to provide valuable insights into the treatment approach for this often‐overlooked yet critical subgroup.

## 2 METHODS

### 2.1 Study selection

**Figure 1:**
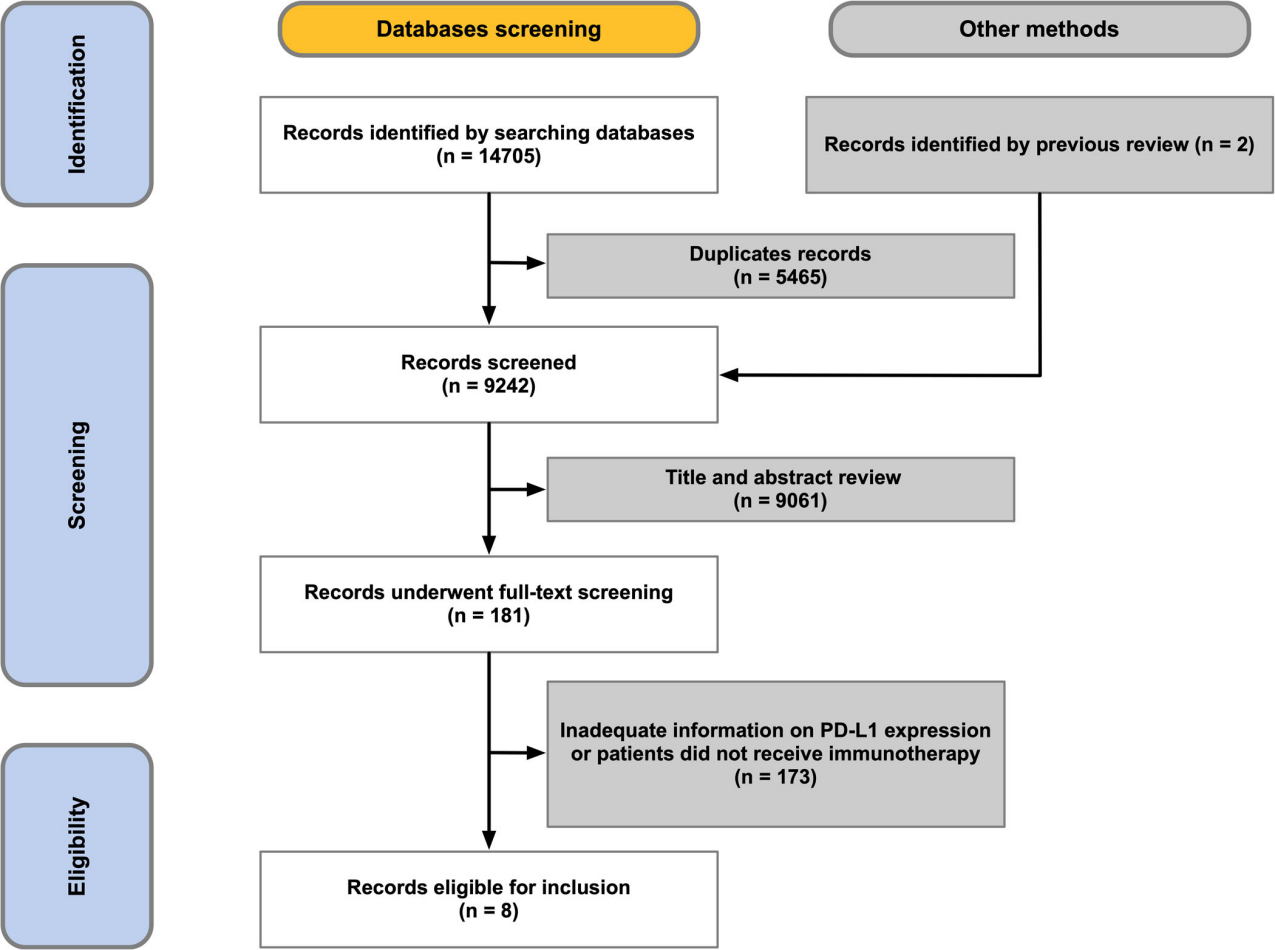
PRISMA diagram. PD‐L1, programmed death ligand 1; PRISMA, preferred reporting items for a systematic review and meta‐analysis.

We conducted a search of studies on Web of Science, Scopus, Pubmed, Cochrane Library, and Embase from inception to January 12, 2023. We searched randomized controlled trials and retrospective studies with advanced CC treated with anti‐PD‐1/PD‐L1 antibodies. The full search string is shown in Supporting Information S1: Table 1. Reference lists of articles selected for full‐text review were scanned for additional relevant studies. A total of 9242 studies were selected after removing overlapping papers. After screening for titles and abstracts, 181 studies underwent full‐text screening, which left eight studies eligible for the final analysis. Regarding studies on the same trial published separately, only the latest and the most relevant study was included. Two individual investigators (W. T. C. and N. Z.) were responsible for the screening process. Disagreements were resolved by consensus. The inclusion criteria were as follows: (1) pathologically identified cancer of the cervix (2) patients were treated with ICIs (anti‐PD‐L1 or anti‐PD‐1 antibody) (3) subgroup survival analysis based on PD‐L1 expression. The process of study selection was demographically shown in Figure 1. Studies that fitted in the following situations were excluded: (1) Patients aged younger than 18 or over 75. (2) Patients who had prior treatment with ICI. The study was conducted according to the Preferred Reporting Items for a Systematic Review and Meta‐analysis (PRISMA) guideline for IPD.^9^ The meta‐analysis was registered with PROSPERO (CRD42023395103).

### 2.2 Data extraction and quality assessment

The following information was extracted from eligible studies: author, publication year, number of patients, treatment, PD‐L1 expression level, the HRs and 95% confidence interval (CI) of OS and PFS, and median follow‐up time. IPD was extracted by using the shiny app IPDfromKM^10^ and the R package KMSubtraction (version 1.0.0.0).^11^ For extracted time‐to‐event data, the Kaplan−Meier (KM) plots were reconstructed and compared to the originally published KM plots. For PD‐L1 negative survival data derived by KMSubtraction, HR was compared to the original article.

### 2.3 Risk of bias assessment

We applied Robins‐I^12^ and Rob 2^13^ to evaluate the risk of bias of included non‐RCT and RCT studies, respectively. Details of the assessment can be found in Supporting Information S1: Figure 1.

### 2.4 Statistical analysis

The primary outcomes of the meta‐analysis were OS and PFS. OS was defined as the time from the inclusion/randomization to death due to any cause. PFS was defined as the time from the inclusion/randomization to disease progression or death from any cause. A pooled analysis based on IPD was carried out by drawing KM curves and conducting log‐rank tests. The Cox regression model was used to evaluate the relative risk, and the HR and 95% CI were reported for the concerned subgroups. A two‐sided *p* < 0.05 was considered as statistically significance. All statistical analysis was conducted in R version 4.1.2.

## 3 RESULTS

### 3.1 Study selection and baseline characteristics

The study selection process is delineated as per the PRISMA guidelines, as depicted in Figure 1. The key attributes of eight enrolled studies (KEYNOTE‐158,^14^ KEYNOTE‐826,^6^ KEYNOTE‐028,^15^ CLAP,^16^ ChekMate‐358,^8^ Tamura et al.,^17^ Empower Cervical 1/GOG‐3016/ENGOT‐cx9,^7^ Xu et al.^18^) are illustrated in Supporting Information S1: Table 2. In the evaluation of PD‐L1 expression, both the CPS and TPS methodologies were employed across the selected studies. Within the cohort, six studies employed CPS, while two studies employed TPS for assessing PD‐L1 expression. Notably, a common threshold of CPS = 1 or TPS = 1% was applied in all the studies for dichotomization. Certain studies did not perform PD‐L1 expression tests on all enrolled patients, with the lowest observed rate of available PD‐L1 expression being 43.05%, as evidenced by Tewari et al. 2022.^7^ The duration of median follow‐up time spanned between 8.6 and 22.0 months. Treatment regimens within the included studies were diverse and could be broadly categorized into three cohorts: ICI (ICI monotherapy), ICI‐OT (ICI combination therapy), and non‐ICI (standard‐of‐care). Furthermore, the term “ICI‐containing” denotes the collective representation of patients who were subjected to either ICI monotherapy or ICI combination therapy.

### 3.2 Reconstruction of time‐to‐event outcomes

**Figure 2:**
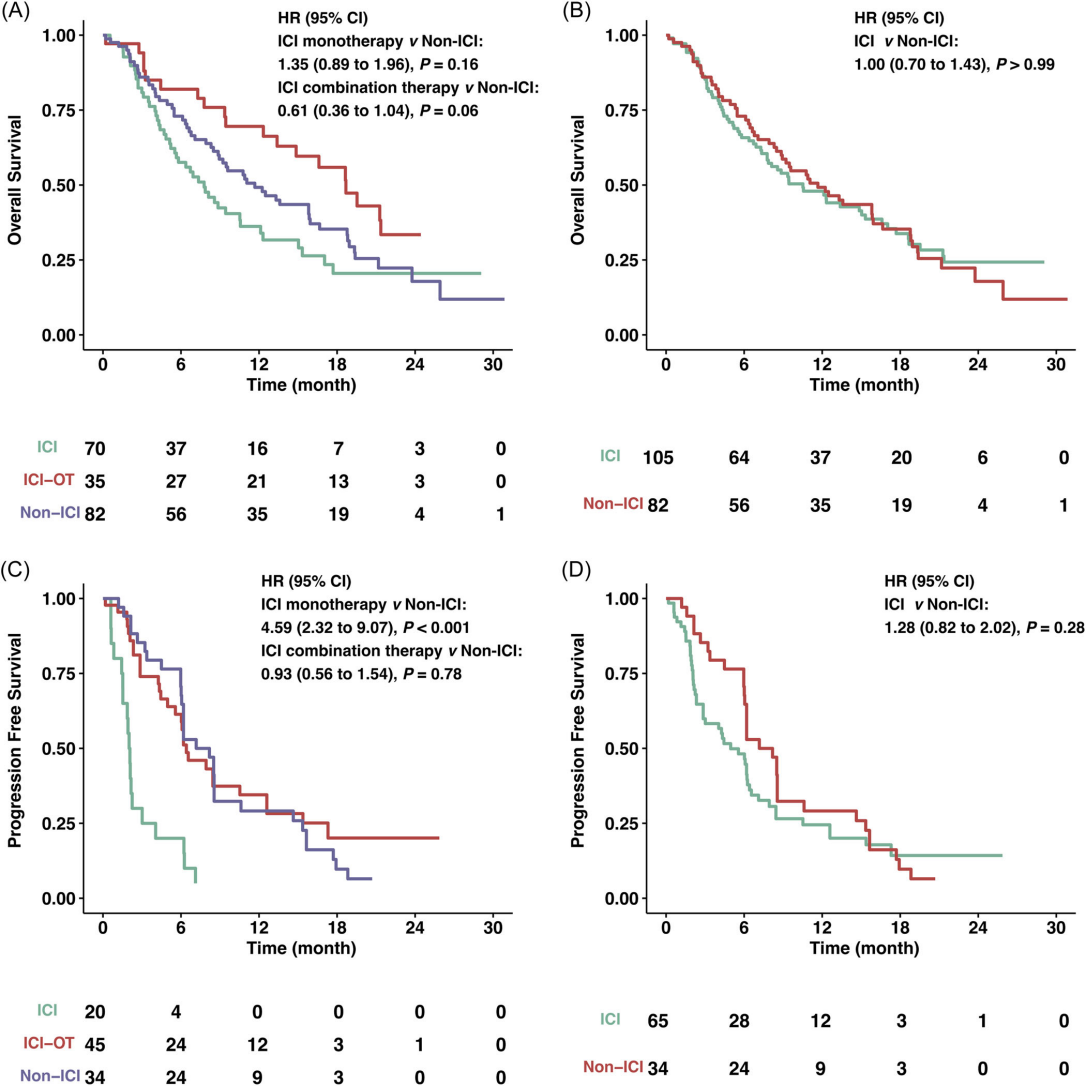
Kaplan−Meier estimates of overall survival (OS) and progression‐free survival (PFS) in low PD‐L1 subgroups. (A, B) OS in low PD‐L1 subgroups stratified by ICI monotherapy, ICI combination therapy, or non‐ICI therapy (A) or ICI‐containing therapy and non‐ICI (B). (C, D) PFS in low PD‐L1 subgroups stratified by ICI monotherapy, ICI combination therapy, or non‐ICI therapy (C) or ICI‐containing therapy and non‐ICI (D). CI, confidence interval; HR, hazard ratio; ICI, immune checkpoint inhibitors; ICI‐OT, ICI combination therapy; PD‐L1, programmed death ligand 1.

To reconstruct time‐to‐event outcomes, a two‐pronged approach was employed in the context of the KEYNOTE‐826 study.^6^ Given the availability of KM plots for OS and PD‐L1 CPS ≥ 1, but not for PD‐L1 CPS < 1, a combination of the IPDfromKM method and KMSubtraction technique was employed. The reconstructed KM plot for PD‐L1 CPS < 1 was depicted in Supporting Information S1: Figure 2. In terms of outcomes, the combination of pembrolizumab and chemotherapy failed to confer advantages in both OS and PFS (OS: HR = 0.99, 95% CI: 0.52−1.86, *p* = 0.97; PFS: HR = 0.83, 95% CI: 0.48−1.42, *p* = 0.49). For other studies, reconstructed KM plots and corresponding HR and 95% CI were shown in Supporting Information S1: Figure 2.

### 3.3 Pooled analysis of survival outcomes in low PD‐L1 expression subgroup

PD‐L1 low expression group encompassed patients with CPS < 1 or TPS < 1%. A pooled analysis of survival outcomes within the subset characterized by low PD‐L1 expression was conducted, incorporating data from 187 patients with reported OS and 99 patients with reported PFS. In the context of OS, the comparison between ICI combination therapy and non‐ICI interventions revealed a noteworthy benefit in favor of the former (HR = 0.61, 95% CI: 0.36−1.04, *p* = 0.06), as depicted in Figure 2A. However, the distinction in OS outcomes between treatments containing ICI and non‐ICI regimens was inconsequential (HR = 1.00, 95% CI: 0.70−1.43, *p* > 0.99), as illustrated in Figure 2B. With respect to PFS, the effect of ICI monotherapy demonstrated a significant detriment to PFS (HR = 4.59, 95% CI: 2.32−9.07, *p* < 0.001), as visualized in Figure 2C. Analogous to the OS analysis, ICI‐containing therapy yielded a similar trend in PFS, signifying its limited impact (HR = 1.28, 95% CI: 0.82−2.02, *p* = 0.28), as exhibited in Figure 2D.

### 3.4 Pooled analysis of survival outcomes in CPS < 1 or TPS < 1% subgroup

**Figure 3:**
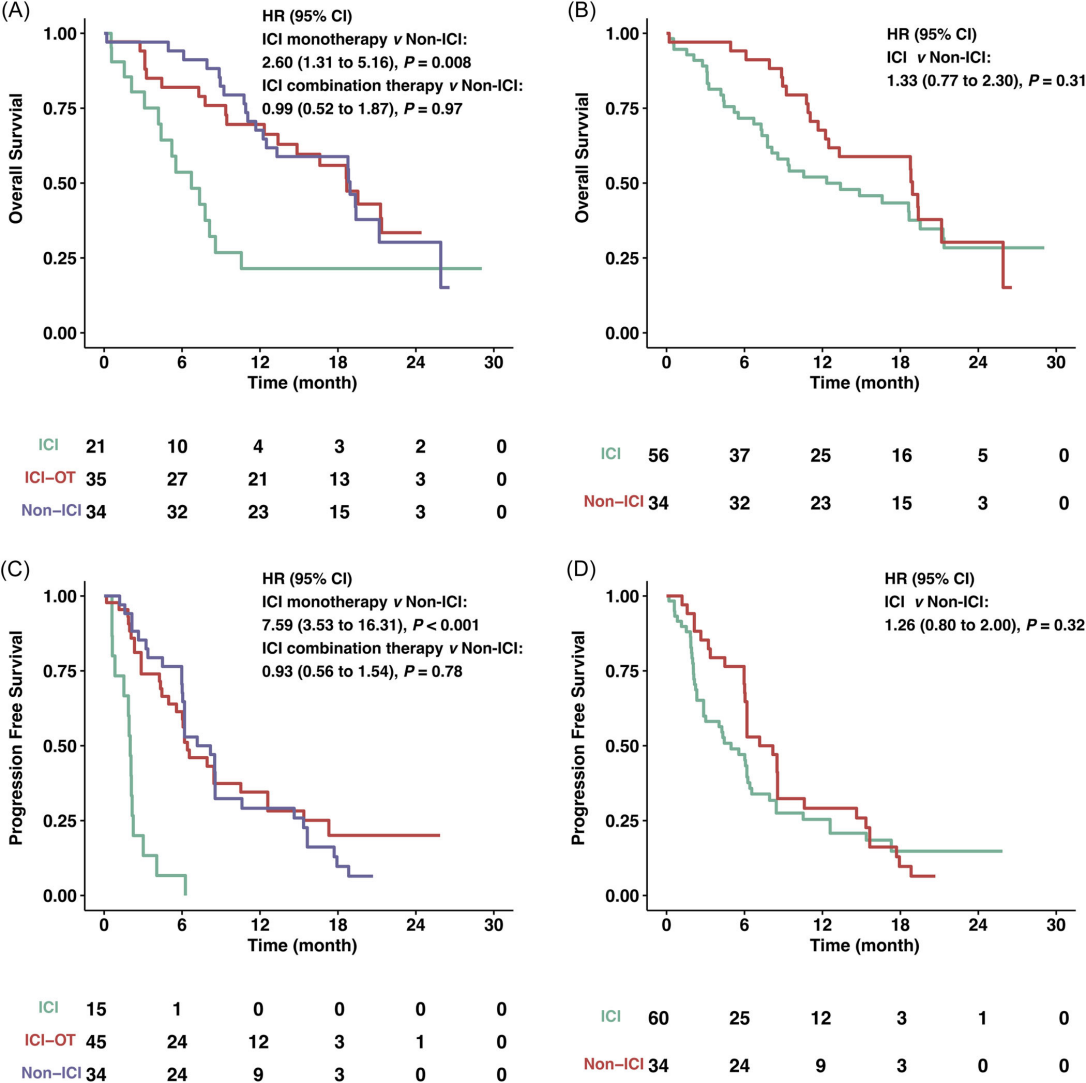
Kaplan−Meier estimates of overall survival (OS) and progression‐free survival (PFS) in low PD‐L1 subgroups, stratified by IHC scoring system. (A, B) OS in CPS < 1 subgroups stratified by ICI monotherapy, ICI combination therapy, or non‐ICI therapy (A) or ICI‐containing therapy and non‐ICI (B). (C, D) PFS in CPS < 1 subgroups stratified by ICI monotherapy, ICI combination therapy, or non‐ICI therapy (C) or ICI‐containing therapy and non‐ICI (D). CI, confidence interval; CPS, combined positive score; HR, hazard ratio; ICI, immune checkpoint inhibitors; ICI‐OT, ICI combination therapy; IHC, immunohistochemistry; PD‐L1, programmed death ligand 1; TPS, tumor proportion score.

To elucidate the differential impact of distinct immunohistochemistry (IHC) scoring systems, we conducted a further analysis to evaluate the efficacy of ICI within the CPS < 1 and TPS < 1% subgroups. Within the CPS < 1 subgroup, the protective influence of ICI combination therapy on OS was less evident compared to the outcomes observed in the overall cohort (HR = 0.99, 95% CI: 0.52−1.87, *p* = 0.97) (Figure 3A). A similar pattern was witnessed in terms of PFS, underscoring the comparable absence of discernible PFS benefits between ICI combination therapy and non‐ICI interventions (HR = 0.93, 95% CI: 0.56−1.54, *p* = 0.78) (Figure 3C). Remarkably, ICI monotherapy was associated with significantly worse OS and PFS (OS: HR = 2.60, 95% CI: 1.31−5.16, *p* = 0.008; PFS: HR = 7.59, 95% CI: 3.53−16.31, *p* < 0.001), as portrayed in Figure 3A,C. Among these findings, ICI monotherapy exhibited the poorest OS outcome (median survival 6.72 months, 95% CI: 4.38 to NA), while the distinction between ICI combination therapy (median survival 18.7 months, 95% CI: 13.4 to NA) and non‐ICI (median survival 18.9 months; 95% CI: 12.5 to NA) was indiscernible.

Regarding ICI‐containing therapy, its impact on both OS and PFS was akin to that of non‐ICI interventions (OS: HR = 1.33, 95% CI: 0.77−2.30, *p* = 0.31; PFS: HR = 1.26, 95% CI: 0.80−2.00, *p* = 0.32), as represented in Figure 3B,D.

In the TPS < 1% subgroup, the analysis was conducted based on two involved studies (Tamura et al.,^17^ and EMPOWER Cervical 1/GOG‐3016/ENGOT‐cx9) that encompassed ICI and chemotherapy regimens. In this context, patients undergoing ICI monotherapy did not exhibit a prolonged OS (HR = 0.83, 95% CI: 0.51–1.35; *p* = 0.46), as visualized in Supporting Information S1: Figure 3. The median survival time for ICI monotherapy was 8.85 months (95% CI: 5.64–15.3), while the corresponding figure for non‐ICI interventions stood at 6.63 months (95% CI: 5.32–15.80).

### 3.5 Pooled analysis of survival outcomes based on all IPD

**Figure 4:**
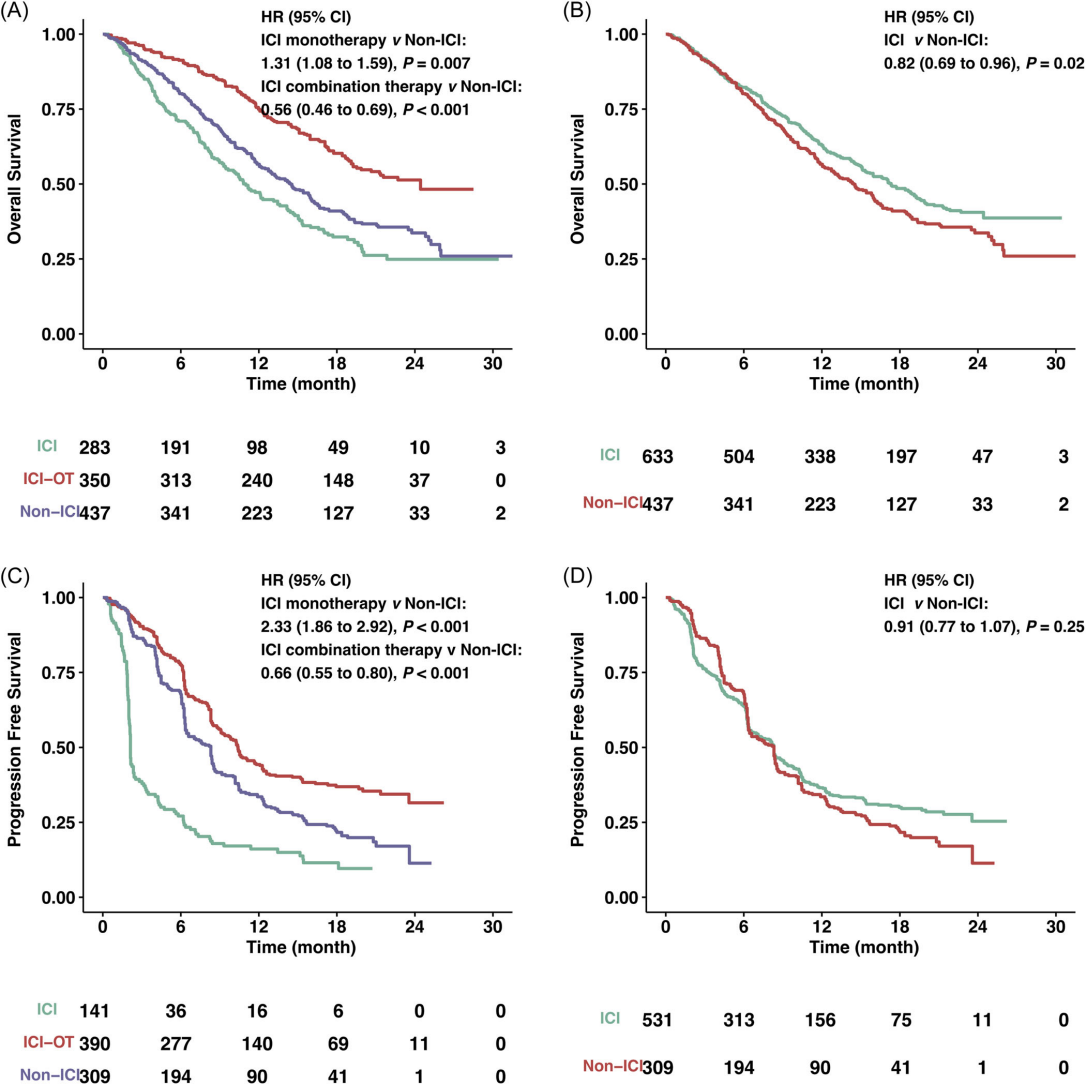
Kaplan–Meier estimates of overall survival (OS) and progression‐free survival (PFS) in the overall cohort. (A, B) OS stratified by ICI monotherapy, ICI combination therapy, or non‐ICI therapy (A) or ICI containing therapy and non‐ICI (B). (C, D) PFS stratified by ICI monotherapy, ICI combination therapy, or non‐ICI therapy (C) or ICI containing therapy and non‐ICI (D). CI, confidence interval; HR, hazard ratio; ICI, immune checkpoint inhibitors; ICI‐OT, ICI combination therapy.

To comprehensively explore the distinct efficacies of therapies in the context of advanced CC, a pooled analysis of OS and PFS was conducted, irrespective of PD‐L1 expression (Figure 4). In terms of OS, it was discerned that among the three subgroups, ICI monotherapy exhibited the least favorable survival outcomes, ICI combination therapy (ICI‐OT) demonstrated the most favorable outcomes, and non‐ICI interventions occupied an intermediate position. Specifically, the HR for ICI‐OT versus non‐ICI was 0.56 (95% CI: 0.46–0.69, *p* < 0.001), while the HR for ICI monotherapy versus non‐ICI was 1.31 (95% CI: 1.08–1.59, *p* = 0.007), as depicted in Figure 4A. The median survival times for these groups were as follows: ICI: 11 months (95% CI: 9.43–13.4), ICI‐OT: 24.4 months (95% CI: 19.5 to NA), and non‐ICI: 14.4 months (95% CI: 12.7–16.0). In addition, ICI‐containing therapy demonstrated OS benefits regardless of PD‐L1 expression when compared with non‐ICI interventions (HR = 0.82, 95% CI: 0.69–0.96, *p* = 0.02), as illustrated in Figure 4B.

With respect to PFS, ICI‐OT showed the most favorable survival outcomes, as illustrated in Figure 4C. The respective median survival times were: ICI: 2.12 months (95% CI: 2.07−2.41), ICI‐OT: 10.4 months (95% CI: 9.09−11.8), and non‐ICI: 8.31 months (95% CI: 6.45−8.51). Notably, ICI‐containing therapy and non‐ICI interventions did not exhibit discernible differences in terms of PFS (HR = 0.91, 95% CI: 0.77−1.07, *p* = 0.25), as shown in Figure 4D.

## 4 DISCUSSION

Our present IPD‐based meta‐analysis divulges insights into the effectiveness of ICI within the spectrum of PD‐L1 negative expression subgroups (CPS < 1 or TPS < 1%) in advanced CC patients. This investigation is notably the first meta‐analysis scrutinizing the efficacy of ICI in the realm of persistent, recurrent, and metastatic CC, with an emphasis on PD‐L1 expression stratification.

The realm of oncology has witnessed an exponential surge in the development of ICI‐based immunotherapy, spurring a multitude of clinical trials testing PD‐1/PD‐L1 inhibitors within the clinical ambit of advanced CC. While concurrent chemo‐radiation remains the mainstay therapy for locally advanced CC, a significant stride was achieved with the FDA's approval of pembrolizumab in combination with platinum chemotherapy for treating PD‐L1 positive recurrent and metastatic CC.^5^ However, the question of whether PD‐1/PD‐L1 inhibitors hold efficacy for PD‐L1 low expression (CPS < 1 or TPS < 1%) patients has remained unanswered, a query that has garnered heightened attention in other malignancies such as melanoma, renal cell carcinoma, non‐small cell lung cancer (NSCLC), and esophageal carcinomas.^19^, ^20^

PD‐L1 expression serves as a predictive marker for the efficacy of immunotherapy. A series of clinical trials have explored the impact of ICIs on advanced CC, categorized by CPS stratification. For instance, the NCT03104699 trial showed responses to balstilimab across both CPS ≥ 1 and CPS < 1 subgroups, albeit with divergent overall response rates (CPS ≥ 1: 20%; CPS < 1: 7.9%).^21^ Similarly, a phase II trial examining balstilimab in conjunction with the anti‐CTLA‐4 inhibitor zalifrelimab demonstrated higher overall response rates in the CPS ≥ 1 subgroup (32.8%) compared to CPS < 1 subgroup (9.1%).^22^ Our study, based on CPS stratification, underscores the superiority of ICI‐OT and non‐ICI interventions over ICI monotherapy in terms of OS and PFS within the CPS < 1 subgroup. However, the determination of the optimal therapeutic strategy between ICI‐OT and non‐ICI in this subgroup necessitates further exploration (ICI‐OT vs. non‐ICI: HR for OS = 0.61, 95% CI: 0.36−1.04, *p* = 0.61; HR for PFS = 0.93, 95% CI: 0.56−1.54, *p* = 0.78). Therefore, it is plausible that a combined therapy approach could potentially surmount the limitations of ICI monotherapy in the context of advanced CC. The interim analysis of the CheckMate‐358 study indicated promising overall response rates (33.3%, 9.1%, 0%, and 57.1% in four different settings) for PD‐L1 negative (TPS < 1%) recurrent/metastatic CC.^8^

Within the TPS < 1% subgroup, consisting of GOG‐3016 and Tamura et al., studies, our analysis demonstrated parity between ICI and non‐ICI interventions with regard to OS (ICI vs. non‐ICI: HR = 0.83; 95% CI: 0.51−1.35), akin to the outcomes of the GOG‐3016 trial (HR = 0.98; 95% CI: 0.59−1.62). The concurrence of our results with those of GOG‐3016 can potentially be attributed to the limited number of eligible studies utilizing TPS as the parameter for PD‐L1 expression measurement.

It is imperative to exercise caution while interpreting the clinical implications of CPS and TPS, the two current methods for assessing PD‐L1 expression through IHC. Both methods present challenges in clinical application due to the lack of a definitive threshold and the heterogeneity observed in the spatial and temporal distribution of PD‐L1.^23^ The superiority of CPS over TPS in identifying ICI responders in CC has been indicated by existing evidence.^24^ Moreover, the high reproducibility of IHC 22C3 pharmDx in determining CPS in CC has been well established.^25^ However, it is essential to recognize that a meticulous evaluation of PD‐L1 expression in CC necessitates multidisciplinary scrutiny, as its expression may vary across different anatomical sites and time points, as evident from studies in NSCLC.^26^

Several limitations should be acknowledged in the context of our study. First, while the IPDfromKM and KMSubtraction methods served as feasible tools for conducting IPD‐based meta‐analysis and integrating patients from unreported groups, it is prudent to recognize that subtle discrepancies may exist between the derived time‐to‐event data and the raw data obtained directly from original investigators. Moreover, the lack of patient‐related clinical data hindered in‐depth analysis. While the acquisition of raw data from these investigators would offer the optimal solution, it remains a challenging endeavor. Second, it is noteworthy that a subset of studies reported both the PD‐L1 status and survival outcomes (OS/PFS), but were not included in our analysis due to the absence of KM curves from which requisite data could be extracted. As a result, only a limited number of patients were included in the analysis, especially when analyzing the PFS group. This limitation underscores the importance of standardizing the reporting of survival outcomes in clinical trials, ensuring that essential data is available for comprehensive meta‐analyses. Therefore, results generated from small samples should be treated with caution. Third, the heterogeneity observed in treatment strategies across present clinical trials focused on CC led to the categorization of interventions into three distinct groups (ICI, ICI‐OT, and non‐ICI). This categorization, while necessary, could potentially introduce variations in the results due to the inherent differences in treatment protocols.

In conclusion, our study shed light on an important consideration in the therapeutic landscape of advanced CC with low PD‐L1 expression. Specifically, our findings suggest that ICI monotherapy may not be the optimal choice as a first‐line therapy in this subset of patients. It is anticipated that the future will see the emergence of additional clinical trials that contribute to a more comprehensive understanding of treatment efficacy in this context.

## AUTHOR CONTRIBUTIONS

**Wutao Chen**: Conceptualization; methodology; software; data curation; investigation; writing—original draft; writing—review and editing; visualization; formal analysis. **Nan Zhang**: Conceptualization; methodology; investigation; writing—original draft; writing—review and editing; validation; formal analysis; funding acquisition. **Zhihong He**: Conceptualization; data curation; writing—original draft; writing—review and editing; formal analysis. **Qing Li**: Project administration; resources. **You Wang**: conceptualization; funding acquisition; methodology; project administration; supervision; writing—original draft; writing—review and editing. **Weihua Lou**: Conceptualization; writing—original draft; writing—review and editing; supervision; project administration; funding acquisition. **Wen Di**: Conceptualization; methodology; formal analysis; funding acquisition; project administration; resources; writing—original draft; writing—review and editing. All authors have read and approved the final version of the manuscript.

## CONFLICT OF INTEREST STATEMENT

The authors declare no conflict of interest.

## TRANSPARENCY STATEMENT

The lead author, You Wang, Weihua Lou, Wen Di affirms that this manuscript is an honest, accurate, and transparent account of the study being reported; that no important aspects of the study have been omitted; and that any discrepancies from the study as planned (and, if relevant, registered) have been explained.

## Supporting information

Supporting information.

## Data Availability

All data generated used to perform analysis in the article are available with a reasonable request to the corresponding author. W. D. had full access to all of the data in this study and takes complete responsibility for the integrity of the data and the accuracy of the data analysis.
